# Novel Prion Protein Gene Polymorphisms in Awassi Sheep in Three Regions of the Fertile Crescent

**DOI:** 10.3390/vetsci10100597

**Published:** 2023-09-29

**Authors:** Faisal S. Rashaydeh, Mehmet A. Yildiz, Abdulrahman S. Alharthi, Hani H. Al-Baadani, Ibrahim A. Alhidary, Hasan Meydan

**Affiliations:** 1 Department of Agricultural Biotechnology, Faculty of Agriculture, Akdeniz University, Antalya 07058, Türkiye; faisalrashaydeh@yahoo.com; 2 Department of Animal Science, Faculty of Agriculture, Ankara University, Ankara 06110, Türkiye; 3 Department of Animal Production, College of Food and Agriculture Science, King Saud University, Riyadh 11451, Saudi Arabia

**Keywords:** Awassi, scrapie, *PRNP* polymorphism, genetic susceptibility, Fertile Crescent

## Abstract

**Simple Summary:**

The Awassi sheep is the most widely bred fat-tail sheep in the Near and Middle East. In this research, a total of 150 healthy Awassi sheep from Türkiye, the Palestinian Authority, and Saudi Arabia were screened for variants in the prion protein gene (*PRNP*). Genotyping Awassi sheep for the prion protein gene allowed us to assess the level of genetic susceptibility to scrapie in studied populations. In addition, twenty-seven amino acid substitutions were found. Eight of them have not been previously reported. Our results on Awassi sheep may assist selection programs that aim to increase the scrapie-resistant genotypes in the populations to reduce the risk of scrapie.

**Abstract:**

Scrapie is a fatal, neurodegenerative disease that affects sheep and goats, and genetic susceptibility to scrapie in sheep is associated with polymorphisms in the prion protein (*PRNP*) gene. The aim of this study is to identify *PRNP* polymorphism in Awassi sheep from Türkiye, the Palestinian Authority, and Saudi Arabia. A total of 150 healthy sheep were genotyped for *PRNP*, using Sanger sequencing. There were seven alleles and eleven genotypes observed based on codons 136, 154, and 171 of *PRNP*. The ARQ allele was predominant in all populations. The most resistant allele to scrapie, ARR, was present in all three regions. The VRQ allele, associated with the highest susceptibility to scrapie, was detected only in Türkiye at a low frequency. In this study, twenty-seven amino acid substitutions were found. Eight of them (R40Q, G65E, H88L, S98T, A118P, S138T, V192F and L250I) have not been previously reported. These data indicate that sheep breeds close to the sheep domestication center have maintained high genetic diversity in the *PRNP* region. Our findings on *PRNP* will provide valuable insights for sheep breeding programs, aiding in the selection of genotypes resistant to scrapie in Türkiye, the Palestinian Authority, and Saudi Arabia.

## 1. Introduction

Scrapie is a neurodegenerative, fatal, and transmissible disease affecting sheep and goats and has been known since 1732 [1]. This disease is a member of the prion disease family, also known as transmissible spongiform encephalopathies (TSEs). Creutzfeldt–Jakob disease (CJD) in humans, chronic wasting disease (CWD) in deer, and bovine spongiform encephalopathy (BSE) in cattle represent other types of TSEs. In these TSE diseases, a neuronal surface glycoprotein, encoded by prion protein gene (*PRNP*), is converted into an abnormal protease-resistant isoform (PrP^Sc^), which accumulates as the infectious agent in the lymphoid tissues and central nervous system [2].

According to many studies on scrapie-affected sheep, resistance or susceptibility to scrapie is strongly associated with polymorphisms of codons 136, 154 and 171 of the *PRNP* [3,4]. It is well-known that the *PRNP* allele with amino acids: alanine (A), arginine (R), and arginine (R) at codons 136, 154 and 171, respectively (short: ARR), is most resistant to classical scrapie, whereas the VRQ allele is highly susceptible. The wild-type allele, ARQ, is associated with moderate susceptibility to scrapie. According to the 2001 NSP (National Scrapie Plan), the fifteen *PRNP* genotypes based on these three codons were divided into five risk groups (R1–R5) related to resistance or susceptibility to natural scrapie [3]. Because of the European Community Regulation (2003) for scrapie in sheep, sheep breeds have been genotyped in many countries, and breeding programs have been implemented to increase the incidence of the resistant allele, ARR, and to decrease the incidence of the susceptible allele, VRQ, in their sheep breeds [3].

Türkiye, Saudi Arabia, and the Palestinian Authority are included in the Fertile Crescent region, which is where the domestication of sheep and other animals originated. As the domestication center, the animals reared in this region have valuable genetic variation [5]. The Awassi is the most widely bred fat-tail sheep in the Near and Middle East [6] and the most common sheep breed in the Palestinian Authority, Iraq, Syria, and the only native breed in Jordan [7]. Until now, genetic variation in *PRNP* has been investigated in Awassi sheep reared in Türkiye and the Palestinian Authority [8,9], but not in Saudi Arabia. Because of the limited studies on scrapie in the Fertile Crescent region and the importance of this region, the sheep reared in these places should be screened for *PRNP* variants and evaluated for the risk of scrapie. The aim of this study is the identification of polymorphisms and possible new variants in *PRNP* and the evaluation of genetic resistance or susceptibility to scrapie in Awassi sheep reared in Türkiye, the Palestinian Authority, and Saudi Arabia.

## 2. Materials and Methods

In this research, a total number of 150 healthy Awassi sheep, (regardless of sex and age), unrelated to each other, were randomly sampled from three different regions (Türkiye, the Palestinian Authority, and Saudi Arabia) and seven different herds, which are state flocks where pure Awassi were reared (Table 1). Blood samples were collected from vena jugularis into 10 mL tubes with EDTA. Genomic DNA was extracted using a salting-out method [10] and stored at −20 °C until PCR assay. All procedures were approved by the Animal Experimentations Local Ethics Board at Akdeniz University.

The *PRNP*, which is 771 bp in length, was amplified using forward (5′ ATG GTG AAA AGC CAC ATA GGC AGT 3′) and reverse (5′ CTA TCC TAC TAT GAG AAA AAT GAG 3′) primers proposed by Sipos et al. [11]. In a final volume of 50 μL, the PCR reactions were set up with the following ingredients: 100 ng DNA, PCR buffer, 2 mM MgCl_2_, 1 U *Taq* polymerase, and 0.2 mM dNTPs, 10 pmol of each primer. The amplification reaction was carried out using an initial denaturation of 5 min at 94 °C, followed by 35 cycles of 45 s at 94 °C, 45 s at 58 °C, and 60 s at 72 °C, and a final extension of 7 min at 72 °C. After the purification of PCR products with the Qiamp Mini Kit (QIAGEN, Valencia, CA, USA), the samples were sequenced. In cases where we encountered difficulties with DNA sequencing results, such as messy or weak peaks, we carried out PCR reactions again, cleaned up the amplicons, and then re-sequenced the samples. Additionally, novel polymorphisms were confirmed by sequencing twice in both directions on DNA to ensure accuracy. We used Finch TV software 1.4.0 (Geospiza Inc., Seattle, WA, USA) to show and check the sequence chromatograms. The sequences of *PRNP* were aligned using MEGA 7 [12]. Genotype and allele frequencies were estimated by counting the number of genes [13] using the following formulas:

$$X_{ij}=\frac{n_{ij}}{n}\;\mathrm{and}\;{\hat{x}}_{i}=\frac{2n_{ii}+\sum n_{ij}}{2n}$$

where *X_ij_* is the frequency of *A_i_A_j_* genotype; *n_ij_* and *n_ii_* are the number of individuals for heterozygous and homozygous genotypes, respectively; ${\hat{x}}_{i}$ is the allele frequency and *n* is the total number of individuals.

## 3. Results

According to ovine *PRNP* sequencing, twenty-seven amino acid substitutions were found: R40Q, G65E, H88L, S98T, W102G, H114Q, A116E, A118P, G127S, G127V, A136V, M137V, S138T, H143R, G145S, N146S, E149K, R154H, R167K, V169G, Q171K, Q171R, Q171H, Q171N, Q189L, V192F, L250I. Eight of these substitutions have not been previously reported: R40Q (CGA → CAA), G65E (GGA → GAA), H88L (CAT → CTT), S98T (AGC → ACC), A118P (GCT → CCT), S138T (AGC → ACC), V192F (GTC → TTC) and L250I (CTC → ATC). In addition to these twenty-seven amino acid substitutions, five synonymous mutations were observed at codons 51 (CGC/CGT), 153 (TAT/TAC), 175 (CAG/CAA), 230 (AGG/CGG), and 237 (CTC/CTG) in this research. All observed amino acid substitutions and synonymous mutations, along with their distributions in Awassi sheep from the Palestinian Authority, Saudi Arabia, and Türkiye, are shown in Table 2.

The observed allele frequencies based on codons 136, 154, and 171 of the *PRNP* in Awassi sheep in the studied sheep populations are summarized in Table 3. Seven alleles were observed: ARR, ARQ, AHQ, ARH, VRQ, ARK and ARN according to the results of this study. The ARQ allele was found as the predominant allele in all regions studied, with frequencies of 0.39, 0.44, and 0.54 in the Palestinian Authority, Saudi Arabia, and Türkiye, respectively. The ARR allele, which is the most resistant to scrapie, was present in all regions studied, with frequencies of 0.34, 0.20, and 0.15 in the Palestinian Authority, Saudi Arabia, and Türkiye, respectively. The VRQ allele, the most susceptible to scrapie, was only detected in Turkish Awassi sheep, with a frequency of 0.01. The AHQ allele was found only in the Palestinian Awassi sheep population. In addition, the ARH allele was found in all regions studied, with frequencies ranging from 0.20 to 0.34. The ARK and ARN alleles, with the lowest frequencies, were found in this study.

The seven alleles resulted in eleven genotype pairs located at codons 136, 154, and 171 of the *PRNP*: ARR/ARR, ARR/ARQ, ARR/ARH, ARR/AHQ, ARH/ARH, ARH/ARQ, ARQ/ARQ, ARH/ARK, ARH/ARN, ARQ/ARK, and VRQ/ARQ (Table 3). The predominant genotype was ARQ/ARQ in Turkish Awassi sheep (0.40), whereas the ARH/ARQ genotype was predominant in the Palestinian Authority and Saudi Arabian Awassi sheep, with frequencies of 0.36 and 0.48, respectively. Both predominant genotypes corresponded to a risk score of R3 according to the National Scrapie Plan (NSP). The most resistant genotype, ARR/ARR, was not found in Saudi Arabian Awassi sheep. The VRQ/ARQ genotype, one of the most susceptible genotypes, was detected in only one Turkish Awassi sheep.

## 4. Discussion

There is both archeological and genetic evidences of sheep domestication in the Fertile Crescent region [5]. The data from the large number of Neolithic human settlements found in this region strongly indicate that it has long been a major and important domestication center for livestock animal species, mainly sheep and goats. Thus, the Awassi sheep reared in this region may play an important role in maintaining the genetic diversity of these populations.

**Figure 1:**
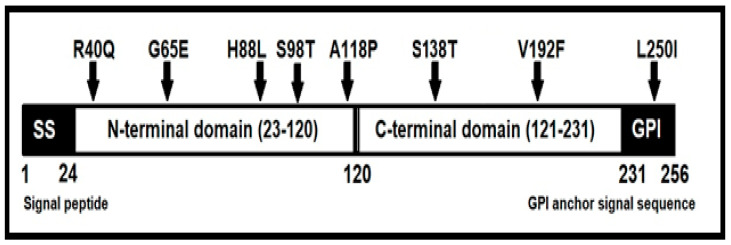
Novel polymorphisms found in this study and their localization in *PRNP*.

According to DNA sequencing results for *PRNP* in Awassi sheep reared in the Palestinian Authority, Saudi Arabia, and Türkiye, twenty-seven amino acid substitutions and five silent mutations were found in this study. Most of them have been previously reported in different countries [14,15,16,17,18,19,20]. The eight amino acid substitutions detected in this study were novel. Among these eight novel polymorphisms, five are located in the N-terminal region and two are found in the C-terminal domain of the *PRNP* protein. In addition, one of them (L250I) was found in the Glycosylphosphatidylinositol (GPI) anchor signal sequence (Figure 1). Two novel polymorphisms found in this study at codon 88 and 98, which are located near the proteinase K cleavage site. The novel polymorphism at codon 118, which is found in the hydrophobic palindrome region, and at codon 127, which was located in the glycine-rich motif, may affect the conformational flexibility of *PRNP* and may introduce a novel form of scrapie in sheep [21].

The GPI-anchor and GPI signal sequence play an important role in protein localization, prion infection, and the disease process [22]. Until now, the GPI anchor site has been assumed to be codon 230 or 231. Based on our 150 *PRNP* sequences, we also predicted the GPI anchor site as codon 231 using online GPI prediction software, NetGPI-1.1 [23]. Although we did not find any polymorphism at codon 231, the new polymorphism at codon 250 in the GPI signal sequence may affect prion infection and the disease process according to the functions of GPI signal sequence. Further studies are needed to determine how the novel variant affects the functions of the prion protein and scrapie susceptibility.

For every country, the identification of *PRNP* polymorphisms in sheep is crucial. Researchers worldwide have genotyped their sheep breeds and developed plans to control scrapie by decreasing the frequency of the susceptible VRQ allele and increasing the frequency of the ARR allele, which is the most resistant allele. In European sheep breeds, *PRNP* polymorphisms have been studied and predicted to be associated with resistance or susceptibility to scrapie through case studies. Additionally, many studies have aimed to investigate ovine *PRNP* polymorphisms in some Asian countries, including Türkiye [9], China [20], India [24], Pakistan [25], Mongolia [26], Iran [27], Jordan [8], Israel [8], Kuwait [28], Nepal [28] and the Palestinian Authority [29]. This study marks the first report identifying *PRNP* polymorphism in Saudi Arabian Awassi sheep. Scrapie control breeding programs in Saudi Arabia, the Palestinian Authority, and Türkiye have not been established to date because there have been no official reports of scrapie-affected sheep in these regions. In this study, we genotyped Awassi sheep for *PRNP* from the studied regions located near the Fertile Crescent and tried to identify which group is at the greatest risk of contracting scrapie.

In this study, the ARQ was found to be the most frequent allele, with frequencies of 0.39, 0.44 and 0.54 for Awassi sheep in the Palestinian Authority, Saudi Arabia, and Türkiye, respectively (Table 1). In previous studies, the frequency of ARQ was estimated at 0.67 and 0.70 for the Palestinian Authority [8] and Turkish [9] Awassi sheep, respectively. The differences between previous results and ours may have arisen due to sampling sheep from different populations within these same countries. The Awassi sheep from all three regions studied do not seem to be resistant to scrapie because the predominant allele is ARQ in these regions. Thus, scrapie breeding programs should be implemented in these regions to increase the incidence of the ARR allele. Most studies conducted in sheep populations across the world have demonstrated that the predominant allele is ARQ [8,9,27,29,30,31,32,33,34]. The ARQ allele is thus regarded as the ovine wild-type allele. Our results seem to support this idea.

The Awassi, a common breed in the Middle Eastern region, had a relatively low frequency of the ARR allele in this study (Table 3). This point is essential because, in the absence of the ARR allele, the only way to develop a breed that is genetically resistant to scrapie would be to introgress the desired gene from different breeds, thereby endangering the original breed. In this study, the ARR allele frequency was estimated as 0.34, 0.20, and 0.15 in the Palestinian Authority, Saudi Arabia, and Türkiye, respectively. The ARR frequency was previously estimated as 0.17 and 0.06 for Awassi sheep in the Palestinian Authority [8] and Türkiye [9], respectively. After comparing the results of previous studies in 2008 and 2012 with the current one, it is apparent that resistance to scrapie in these two regions has been increasing over the last decades. The high-risk allele to scrapie, VRQ, was detected in only Turkish Awassi sheep at a low frequency (0.01). The result of this study is very similar to a previous study in Turkish Awassi sheep [9]. In a previous study [8], the VRQ allele was also not found in Palestinian Awassi sheep. The ARH allele was found in all regions studied, with frequencies ranging from 0.20 to 0.34, and its frequency was estimated 27.6% on average. The ARH allele was the second most common allele in this study. In addition, The ARK and ARN alleles, not included in any scrapie risk group by the NSP, were detected but at a very low frequency. This was also the first time that these alleles were detected in the Palestinian Authority and Saudi Arabia.

Our findings showed that the ARQ/ARQ genotype was found predominant (0.40) for Turkish Awassi sheep in this study (Table 3). This observation is coherent with the previous study (0.53) for Awassi sheep in Türkiye [9] as well as reports on other breeds in different countries [21,25,28,32,35,36]. In contrast to Turkish Awassi sheep, the ARQ/ARH genotype was predominant in Palestinian and Saudi Arabian Awassi sheep, with frequencies of 0.36 and 0.48, respectively. Interestingly, for Palestinian Awassi sheep, the ARQ/ARH genotype was not reported in previous studies [8,29]. The frequency of the ARQ/ARQ geotype was estimated as 0.12 for Palestinian Awassi sheep in this study, while it was previously found to be five times higher at 0.61 [8] and four times higher at 0.52 [29]. For Palestinian Awassi sheep, another interesting result was that the most resistant genotype, ARR/ARR, was detected with a frequency of 0.20, whereas it was not found in previous studies [8,29]. According to these interesting differences between our study and previous reports, it may be proposed that the resistance to scrapie has increased over the past decade. However, in this study, we had a small sample size from the Palestinian Authority. For that reason, further studies with more animals are needed to confirm this suggestion conclusively. The ARR/ARR genotype was found in Awassi sheep in the Palestinian Authority (0.20) and Türkiye (0.06), but not in Saudi Arabia. The absence of the ARR/ARR genotype poses one of the primary challenges for scrapie breeding programs aimed at increasing the incidence of the ARR allele. Thus, the scrapie breeding programs should be established for Saudi Arabian Awassi sheep. One of the most susceptible genotypes, a member of the R5 risk group, VRQ/ARQ, was detected in only Turkish Awassi sheep, but at a very low frequency (0.02). Similarly, the frequency of the VRQ/ARQ genotype was estimated as 0.01 in a previous study [9]. The ARQ/ARK genotypes, not included in any risk group by NSP, were detected in only Turkish Awassi sheep, whereas the ARH/ARK genotype was found in Palestinian and Saudi Arabian Awassi sheep. In previous studies, the identification of one scrapie case in a sheep carrying the ARK allele showed that this allele did not provide full resistance against scrapie. The effect of ARQ/ARK genotypes on susceptibility to scrapie has not been fully studied, but a report of a scrapie-positive sheep with the genotype ARK/ARH suggests that it may not confer resistance [37,38].

It was found that the Awassi sheep in the studied regions belonged to the R3 risk group according to NSP. The incidence of the R3 risk group was calculated as 48%, 56%, and 72% in Palestinian, Saudi Arabian, and Turkish sheep breeds, respectively. The most resistant group, R1, was not found in Saudi Arabian Awassi sheep. Therefore, such breeding programs should be established for this country to increase the number of sheep with the ARR/ARR genotype. The highest susceptible risk group, R5, was observed in only one Turkish Awassi sheep, in a heterozygous state (VRQ/ARQ). The prevalence of the R3 group in the Palestinian Authority, susceptible to scrapie, was estimated at 0.69 and 0.58 by Gootwine et al. [8] and Alsayed et al. [29], respectively. In this study, the prevalence of the R3 risk group was calculated to be lower (0.48), similar to previous studies in the Palestinian Authority. In addition, the most resistance group, R1, has not been previously detected. When comparing our results with previous studies, it can be suggested that the increasing occurrence of resistant genotypes to scrapie in Palestinian Awassi sheep is resulting in the increased prevalence of R1 and decreasing prevalence of R3 groups.

Over the past decade, an average of about 25% of ovine ARQ alleles were reported to have additional polymorphisms, and fewer polymorphisms have been recognized for ARR, VRQ, and AHQ alleles. Generally, additional polymorphisms were found in association with the haplotype ARQ/ARQ, which is considered the wild-type haplotype [20]. In this study, all additional polymorphisms except A118P, M137V, and G145S were detected with different haplotypes from ARQ/ARQ (Table 4). For example, H114Q was found with six different haplotypes, including ARQ/ARQ, ARR/ARR, ARQ/ARH, ARR/ARQ, ARH/ARK, and ARH/ARN. Interestingly, nine additional polymorphisms (A116E, G127S, G127V, S138T, H143R, E149K, R167K, V169G, and V192F) were not associated with the ARQ/ARQ haplotype. It can be concluded that the reason of these interesting results may be because of the sampling from regions near the domestication center in this study. According to many studies, animals in these regions have increased genetic variation compared to animals reared in other regions. All additional polymorphisms identified to date either enhance or reduce susceptibility to scrapie or lengthen/shorten the incubation period. In addition, some polymorphisms have not yet been assigned to specific risk categories due to a lack of available data. Thus, although we identified some additional polymorphisms of *PRNP*, we could not identify their effects as we had no scrapie-affected sheep for analysis. There have been no recorded scrapie cases in sheep breeds in the Fertile Crescent region, which includes Türkiye, the Palestinian Authority, and Saudi Arabia. It may be concluded that *PRNP* genotypes of animals from these regions are contributing to conferring resistance in sheep against scrapie. Further studies are needed for this conclusion in different sheep breeds from these regions, which are close to the sheep domestication center.

## 5. Conclusions

In this study, eight novel polymorphisms were detected in Awassi sheep reared in Türkiye, the Palestinian Authority, and Saudi Arabia. It was suggested by Kijas et al. [39] that domestication occurred from a broad genetic base. Our study about *PRNP* in Awassi sheep supports this suggestion, as it reveals high genetic diversity in Awassi breeds from the Fertile Crescent region. According to the findings of this study, the Awassi sheep sampled were classified into risk group R3, followed by R2. Because of the lack of resistant genotypes and potential protective alleles in the Awassi sheep, it can be concluded that the populations under study are genetically less resistant to scrapie. Our results regarding *PRNP* polymorphisms in Awassi sheep will help the scrapie breeding programs aiming to increase the prevalence of resistant genotypes in sheep populations and thereby reduce the risk of scrapie.

## Figures and Tables

**Table 1:** Sampling localities and sample size.

Region	Sample Size	Location/Herd
Türkiye	50	Şanlıurfa (*n* = 25) Gaziantep (*n* = 25)
Palestinian Authority	50	Jenin (*n* = 15) Jericho (*n* = 15) Hebron (*n* = 20)
Saudi Arabia	50	Riyadh (*n* = 25) Taif (*n* = 25)

**Table 2:** Number of various *PRNP* polymorphisms and their occurrence in studied sheep populations.

Variation			Breed			
DNA	Codon	Protein	Palestinian Authority	S. Arabia	Türkiye	Total
119G → A	CGA/CAA	R40Q*	5	10	-	15
153C → T	CGC/CGT	Silent	-	2	-	2
194G → A	GGA/GAA	G65E*	-	1	-	1
263A → T	CAT/CTT	H88L*	-	2	-	2
293G → C	AGC/ACC	S98T*	4	9	-	13
304T → G	TGG/GGG	W102G	5	11	-	16
342T → G	CAT/CAG	H114Q	6	7	-	13
347A → G	GCA/GAA	A116E	-	3	-	3
352G → C	GCT/CCT	A118P*	1	-	-	1
379G → A	GGC/AGC	G127S	2	3	2	7
380G → T	GGC/GTC	G127V	1	1	1	3
407C → T	GCC/GTC	A136V	-	-	1	1
409A → G	ATG/GTG	M137V	-	-	2	2
413G → C	AGC/ACC	S138T*	2	3	-	5
428A → G	CAT/CGT	H143R	-	-	2	2
433G → A	GGC/AGC	G145S	-	-	1	1
437A → G	AAT/AGT	N146S	1	3	11	15
445G → A	GAG/AAG	E149K	1	1	-	2
459T → C	TAT/TAC	Silent	2	-	-	2
461G → A	CGT/CAT	R154H	4	-	-	4
500G → A	AGA/AAA	R167K	5	8	-	13
506T → G	GTG/GGG	V169G	3	6	-	9
511C → A	CAG/AAG	Q171K	2	2	1	5
512A → G	CAG/CGG	Q171R	24	20	12	56
513G → T	CAG/CAT	Q171H	20	34	21	75
511C → A 513G → T	CAG/AAT	Q171N	1	-	-	1
525G → A	CAG/CAA	Silent	4	2	-	6
566A → T	CAA/CTA	Q189L	-	-	5	5
574G → T	GTC/TTC	V192F*	-	2	-	2
691C → A	AGG/CGG	Silent	4	8	-	12
711C → G	CTC/CTG	Silent	4	8	-	12
748C → A	CTC/ATC	L250I*	5	7	-	12

Numbers represent occurrence of polymorphism, either in the heterozygous or homozygous state.

**Table 3:** Genotype and allele frequencies of the *PRNP* in the studied Awassi sheep populations.

NSP *		Palestinian Authority *n* = 50	Saudi Arabia *n* = 50	Türkiye *n* = 50
Genotype frequency				
R1	ARR/ARR	0.20 (10)	0	0.06 (3)
R2	ARR/AHQ	0.08 (4)	0	0
R2	ARR/ARH	0	0.16 (8)	0.10 (5)
R2	ARR/ARQ	0.20 (10)	0.24 (12)	0.08 (4)
R3	ARH/ARH	0	0	0.16 (8)
R3	ARQ/ARH	0.34 (17)	0.48 (24)	0.16 (8)
R3	ARQ/ARQ	0.12 (6)	0.08 (4)	0.40 (20)
R5	VRQ/ARQ	0	0	0.02 (1)
**	ARH/ARK	0.04 (2)	0.04 (2)	0
**	ARQ/ARK	0	0	0.02 (1)
**	ARH/ARN	0.02 (1)	0	0
Allele frequency				
	ARR	0.34	0.20	0.15
	AHQ	0.04	0	0
	ARQ	0.39	0.44	0.54
	ARH	0.20	0.34	0.29
	VRQ	0	0	0.01
	ARK	0.02	0.02	0.01
	ARN	0.01	0	0

* Risk groups suggested by National Scrapie Plan (NSP). ** Not included in any group by NSP. The numbers in parentheses show number of individual genotypes.

**Table 4:** Additional polymorphisms and their linked haplotypes of *PRNP* in studied Awassi sheep populations.

Variations	Haplotypes Based on Codons 136/154/171
R40Q	*ARQ/ARQ*, ARQ/ARH, ARR/AHQ, ARH/ARN
G65E	*ARQ/ARQ*, ARR/ARQ
H88L	*ARQ/ARQ*, ARR/ARQ
S98T	*ARQ/ARQ*, ARQ/ARH, ARR/AHQ, ARR/ARH
W102G	*ARQ/ARQ*, ARQ/ARH, ARR/ARQ, ARH/ARK, ARR/ARH,
H114Q	*ARQ/ARQ*, ARR/ARR, ARQ/ARH, ARR/ARQ, ARH/ARK, ARH/ARN
A116E	ARR/ARQ
A118P	*ARQ/ARQ*
G127S	ARQ/ARH, ARR/ARQ
G127V	ARQ/ARH, ARH/ARH
M137V	*ARQ/ARQ*
S138T	ARR/ARR, ARQ/ARH, ARR/ARQ, ARR/ARH
H143R	ARQ/ARH
G145S	*ARQ/ARQ*
N146S	*ARQ/ARQ*, ARQ/ARH, ARH/ARH, ARR/ARQ
E149K	ARH/ARK, ARQ/ARH
R167K	ARQ/ARH, ARH/ARK, ARR/ARQ, ARR/ARH
V169G	ARQ/ARH, ARR/ARQ, ARR/ARH
Q189L	*ARQ/ARQ*, ARR/ARH, ARH/ARH, ARQ/ARH
V192F	ARR/ARQ
L250I	*ARQ/ARQ*, ARR/ARQ, ARQ/ARH, ARR/ARH

The wild-type haplotype, ARQ/ARQ, was italicized.

## Data Availability

The data presented in this study are available on request from corresponding author.
